# The case for home based telehealth in pediatric palliative care: a systematic review

**DOI:** 10.1186/1472-684X-12-4

**Published:** 2013-02-01

**Authors:** Natalie Bradford, Nigel R Armfield, Jeanine Young, Anthony C Smith

**Affiliations:** 1 Centre for Online Health, School of Medicine, The University of Queensland, Brisbane, Australia; 2 Queensland Children’s Cancer Centre, Royal Children’s Hospital, Queensland, Australia; 3 Queensland Children’s Medical Research Institute, The University of Queensland, Brisbane, Australia; 4 Nursing Research, Royal Children’s Hospital, Brisbane, Australia

**Keywords:** Palliative care, Pediatric, Telehealth, Home care

## Abstract

**Background:**

Over the last decade technology has rapidly changed the ability to provide home telehealth services. At the same time, pediatric palliative care has developed as a small, but distinct speciality. Understanding the experiences of providing home telehealth services in pediatric palliative care is therefore important.

**Methods:**

A literature review was undertaken to identify and critically appraise published work relevant to the area. Studies were identified by searching the electronic databases Medline, CINAHL and Google Scholar. The reference list of each paper was also inspected to identify any further studies.

**Results:**

There were 33 studies that met the inclusion criteria of which only six were pediatric focussed. Outcome measures included effects on quality of life and anxiety, substitution of home visits, economic factors, barriers, feasibility, acceptability, satisfaction and readiness for telehealth. While studies generally identified benefits of using home telehealth in palliative care, the utilisation of home telehealth programs was limited by numerous challenges.

**Conclusion:**

Research in this area is challenging; ethical issues and logistical factors such as recruitment and attrition because of patient death make determining effectiveness of telehealth interventions difficult. Future research in home telehealth for the pediatric palliative care population should focus on the factors that influence acceptance of telehealth applications, including goals of care, access to alternative modes of care, perceived need for care, and comfort with using technology.

## Background

Palliative care is defined as a philosophy of care, which aims to holistically address concerns affecting quality of life that arise when a person is diagnosed with a life threatening illness. These concerns include physical and psychological symptoms as well as social and spiritual issues [1]. Palliative care includes end of life care, however particularly in pediatrics, palliative care can also be delivered alongside curative or treatment orientated care. Pediatric palliative care focuses on providing the best possible quality of life for infants and children whose illness make it unlikely that they will survive into adulthood.

While the principles of adult based palliative care are relevant to the care of children, pediatric palliative care is different in some important respects. For instance, there are a wider range of conditions seen, including congenital abnormalities and rare metabolic illnesses, which require care over many years. Care of the whole family is needed, with particular attention to parents, siblings and grandparents. Some conditions may be hereditary, with more than one child in the family affected. Finally most clinicians are less experienced with the conditions and palliative care needs of a child, and care is often led by tertiary specialist teams [2].

Many families prefer the option of home care as opposed to care in a facility as it decreases the interruption to normal everyday life events, and maintains quality of life for the family [3]. For families of children with complex medical needs however, caring at home presents a challenge as clinicians are available on a visiting only basis, and family members must otherwise manage care situations themselves. Families rely on the information, advice and support provided by clinicians [4]; effective communication is therefore crucial.

Telehealth has been proposed as a solution for increasing access to health care services when separated by geography, circumstance, or time by facilitating real time synchronous communication [5]. For families who wish to care for a loved one at home during the palliative phase, telehealth applications may present an option for communicating and exchanging information with health care teams. However, despite advances in technology which has significantly improved the ability to provide home telehealth services, the uptake of applications has been slow, and the full potential of the modality has not been realised [6]. The purpose of this study was to review the evidence for home-based telehealth in palliative care, particularly in pediatrics.

## Methods

### Search strategy

The literature search was performed using the electronic databases Medline and the Cumulative Index to Nursing and Allied Health Literature (CINAHL). The databases were last searched on the 22^nd^ February 2012.

Medline was searched with the MeSH terms:

Palliative care AND (telehealth OR telemedicine OR remote consultation).

The CINAHL database was searched with the Medical Major (MM) terms

Telehealth AND Palliative care

As the initial database searches did not yield many relevant articles, a second pass of the literature was taken and the search was expanded to include hand searching of referenced articles, Google Scholar, and SmartText searching on CINAHL using the original terms as well as the terms ‘telehomecare’, ‘telepediatrics’, ‘video-conferencing’, AND ‘palliative care’, as well as the term ‘telehospice’.

Titles of all articles were reviewed and, if considered relevant, abstracts were then examined.

Each article included in the review was evaluated for validity using the appropriate grading tool for its study design, from a suite of tools designed by the Critical Appraisal Skills Programme (CASP) [7]. Depending on study design, between 10 and 12 criteria were assesed as being met (Y), unable to be met (U), or not meeting criteria. Each article was then summarized with regard to: study design, the number of articles/participants, validity, and the study outcomes. Study outcomes included effects on quality of life and anxiety; substitution of home visits; economic considerations; readiness for telehealth; and the barriers, feasibility, acceptability and satisfaction of telehealth. The principle criterion for determining the effectiveness of an intervention has been defined as the ability to produce more good than harm [8]. Using this definition along with the appraised (CASP) [7] validity score of each study, studies were coded based on whether the findings were i) supportive, ii) inconclusive, or iii) unsupportive of the use of telehealth to provide palliative care in the home. If a study reported outcomes supportive of telehealth, but did not satisfy four or more CASP categories of validity, they were categorized as partially supportive. Following appraisal of the studies, a practical framework was developed to understand the relationships between identified factors in the studies.

### Inclusion criteria

The use of telehealth for home-based palliative care is a relatively new area; therefore all studies and published literature of any study design were examined.

Articles, published in the English language, which described or evaluated the use of real-time telehealth for providing palliative care in the home setting, were eligible for inclusion. The primary aim of the review was to identify and appraise pediatric applications, however adult focussed studies were included as findings may have relevance to the care of children.

Studies were excluded if they did not meet the inclusion criteria or were primarily concerned with aspects of informatics and technology rather than patient care. Studies that described asynchronous communication, e.g. reminder systems; and editorial articles and letters not reporting original research were also excluded from review.

## Results

**Figure 1:**
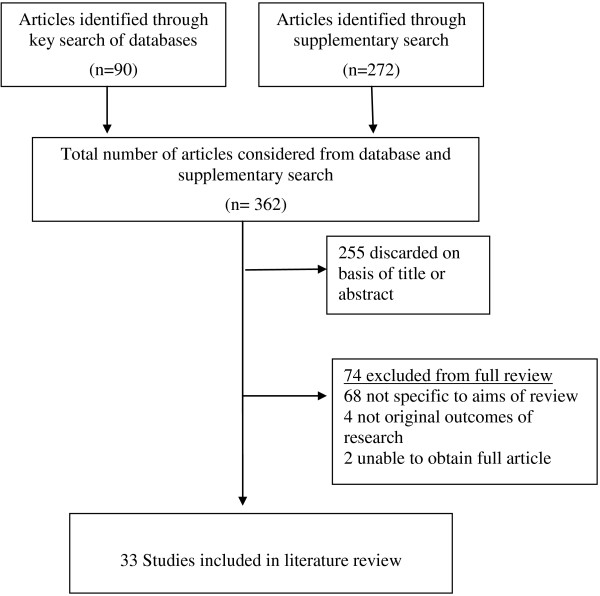
Search flow diagram.

The search of the MEDLINE database yielded a total of 64 articles, and the CINAHL database an additional 26. The supplementary search resulted in a further 272 articles for consideration. Following review of article titles and abstracts, 255 articles were subsequently discarded. After examination a further 74 were excluded as they were not specific to the aims of this review, and two articles could not be obtained, resulting in 33 articles appropriate for formal review (see Figure 1).

### Study design

Table 1 summarises the reviewed articles by study design and participant numbers. Articles that were pediatric focussed are presented alongside adult focused studies. Tables 2 and 3 presents the appraisal of each study used to determine study validity.

**Table 1:** Telehealth studies in palliative care: article study design and participant numbers

**Study design**	**Number of adult studies**	**Number of pediatric studies**	**Number of participants**
Systematic/ literature review	4	-	26-138 papers reviewed
*Randomised Controlled Trials*			
RCT	1	1	27-44
RCT (pilot)	1		12-30
Abandoned RCT		1	-
*Other quantitative designs*			
Cohort	1	1	12-63
Retrospective chart review	2		345-597 charts
Cost comparison	1		3 month period
Analysis of survey	2		6-160
Mixed methods	2		25-68
*Qualitative*			
Focus Group/Interviews	9	1	6-190
Case Study	4	2	1-3
*Total*	*27*	*6*	

**Table 2:** **Appraisal of studies by study design using CASP **[7 ]**tools**

**Study design type**	**Article number [Ref]**	**Did review address a clearly focussed question?**	**Did authors look for appropriate sorts of papers?**	**Were important relevant studies included?**	**Has quality of studies been assessed**	**Was it reason-able to combine results?**	**Are overall results clear?**	**How precise are results**	**Can results be generalised**	**Were all important outcomes considered**			**Is study supportive of the intervention?**	**Validity score**

**Review**	1. [38]	Y	Y	Y	Y	Y	Y	n/a	Y	Y			Y	8/8
	2. [41]	Y	N	N	Y	N	Y	n/a	N	Y			Y	4/8
	3. [40]	Y	Y	Y	N	Y	Y	n/a	N	Y			Y	6/8
	4. [39]	Y	Y	Y	U	Y	Y	n/a	Y	Y			Y	7/8
**RCT**		**Did trial address a clearly focussed issue?**	**Were participant randomised?**	**Where all patients accounted for at conclusion?**	**Was the study blinded?**	**Were groups similar at the start of the trial?**	**Were groups treated equally aside from intervention?**	**Was the effect significant?**	**Was the effect measured with precision?**	**Is study general-isible**	**Were all out-comes consider-ed**		**Is the study supportive of the intervention?**	
	5. [42]	Y	Y	Y	N	Y	Y	Y	Y	Y	Y		Y	9/10
	6. [45]	Y	N	Y	N	Y	Y	n/a	n/a	N	n/a		Y	5/8
	7. [10]	Y	Y	Y	N	Y	Y	n/a	n/a	N	U		Y	6/10
	8. [3]	Y	Y	Y	N	Y	Y	Y	Y	Y	Y		Y	9/10
**Other quant** **itative design**		**Did the study address a clearly focussed issue?**	**Did the authors use appropriate methods to answer their questions?**	**Acceptable recruitment?**	**Was exposure measured to minimize bias?**	**Was the outcome measured to minimise bias?**	**Have the authors identified all confounding factors?**	**Was follow-up com-plete?**	**Are the results statistic-ally significant?**	**Are the results plausible?**	**Are the results genera-lisible**	**Do the results fit with other evidence?**	**Is the study supportive of the intervention?**	
	9. [11]	Y	Y	Y	Y	U	U	Y	N	Y	Y	Y	Y	8/11
	10. [15]	Y	Y	Y	Y	Y	Y	n/a	n/a	Y	Y	Y	Y	9/11
	11. [16]	y	Y	Y	Y	U	U	n/a	n/a	Y	Y	Y	Y	7/11
	12. [36]	Y	Y	U	Y	Y	U	n/a	n/a	Y	N	Y	Y	7/11
	13. [13]	Y	Y	U	Y	U	Y	n/a	n/a	Y	N	Y	U	7/11
	14. [28]	Y	N	Y	Y	N	N	Y	Y	Y	N	Y	Y	7/11
	15. [24]	Y	Y	Y	Y	Y	Y	Y	Y	Y	Y	Y	U	11/11

*Y*= yes, *N*= no, *U*= unclear, *n/a*= not applicable.

**Table 3:** **Appraisal of qualitative studies using CASP **[7 ]**tools**

**Qualitative studies**	**Article number [Ref]**	**Was there a clear statement of the aims of the research?**	**Is qualitative methodology appropriate?**	**Was design appropriate to address the aims?**	**Was the recruitment strategy appropriate to match aims?**	**Were the data collected in a way that addresses the research issue?**	**Has the relationship between researcher and participants been adequately considered?**	**Have ethical issues been taken into consideration?**	**Was the data analysis sufficiently rigorous?**	**Is there a clear statement of findings?**	**Is the research valuable?**	**Is the study supportive of the intervention**	**Validity score**
	16. [14]	Y	Y	Y	U	Y	Y	Y	U	Y	Y	Y	8/10
	17. [25]	Y	Y	Y	Y	Y	Y	Y	Y	Y	Y	Y	10/10
	18. [17]	N	Y	U	Y	U	U	Y	U	Y	Y	Y	5/10
	19. [18]	Y	Y	Y	Y	U	Y	Y	U	Y	Y	Y	8/10
	20. [30]	Y	Y	Y	U	Y	U	U	U	Y	Y	Y	7/10
	21. [19]	Y	Y	Y	Y	U	U	Y	U	Y	Y	Y	7/10
	22. [20]	Y	Y	Y	Y	Y	Y	Y	U	Y	Y	Y	9/10
	23. [26]	Y	Y	Y	Y	Y	U	Y	Y	Y	Y	Y	9/10
	24. [22]	Y	Y	U	Y	U	Y	Y	U	Y	Y	Y	7/10
	25. [21]	Y	Y	Y	Y	Y	Y	Y	Y	Y	Y	Y	10/10
	26. [27]	Y	Y	Y	Y	Y	Y	Y	Y	Y	Y	Y	10/10
	27. [9]	Y	Y	Y	Y	Y	Y	Y	Y	Y	Y	Y	10/10
	28. [37]	Y	Y	Y	Y	Y	Y	Y	Y	Y	Y	Y	10/10
	29. [23]	Y	Y	Y	Y	Y	Y	Y	Y	Y	Y	Y	10/10
	30. [31]	Y	Y	Y	Y	Y	Y	Y	Y	Y	Y	Y	10/10
	31. [29]	Y	Y	Y	Y	Y	Y	Y	Y	Y	Y	U	10/10
	32. [32]	Y	Y	Y	Y	Y	Y	Y	Y	Y	Y	U	10/10
	33. [33]	Y	Y	Y	Y	Y	Y	Y	Y	Y	Y	U	10/10

*Y*= yes, *N*= no, *U*= unclear, *n/a*= not applicable.

### Participants

Participants in the primary studies included in this review were either patients/caregivers receiving palliative care [3,9-23], health professionals providing palliative care [24-29] or both [30,31]. The other articles were either descriptive reports regarding the provision, predictors, costs, barriers and ethical considerations regarding telehealth services for palliative care [32-37], or systematic/literature reviews [38-40].

### Interventions

Table 4 details individual study information. Most studies described the use of synchronous videoconferencing to the home to support and patient and their caregiver during palliation, or at a time when complex medical interventions were being delivered. In most cases, dedicated videoconferencing equipment (e.g. a videophone) was installed in a patient’s home.

**Table 4:** Articles categorised by study design, validity and supportiveness of home telehealth

**Article number**	**Study design**	**Author and Year**	**Study population**	**Validity as determined by CASP tool**	**Conclusions for study support for home telehealth-based on validity and study findings**
1	Review	Bensink, Hailey et al. 2006 [38]	138 studies (only 8 related to ‘videophones’)	8/8	Supportive: Common theme that a lot is written about its potential, but little clinical research and evaluation undertaken
2	Review	Oliver, Demiris et al. 2012 [41]	26 articles	4/8	Partially Supportive: Concerns with study validity, outcome reported as supportive. Acknowledged researcher bias in the field, but review limited to ‘hospice’ no palliative care studies included. Evidence base growing and shows lower to medium strength evidence. More RCTs required
3	Review	Kidd, Cayless et al. 2011 [40]	21 articles	6/8	Supportive: Telehealth is acceptable to professionals and clinicians, and able to advance the borders of accessible care. Lack of evidenced based research for telehealth in palliative care in the UK
4	Review	Gaikwad and Warren 2009 [39]	27 articles	7/8	Supportive: Videoconferencing shown to reduce unplanned admissions, decrease health utilisation, but more studies needed to assess benefit with evidence based outcomes
5	RCT	Hebert, Jansen et al. 2006 [42]	Planned 320 adult	10/10	Supportive: Flexibility required running a RCT in pall care and telemedicine. Telehealth able to achieve comparable results to face to face visits, but not likely to be used due to external factors such as changes to routines and readiness to use telehealth
			palliative care patients		
			- 44 recruited		
6	RCT	Bensink, Armfield et al. 2009 [44]	12 pediatric oncology palliative care families	5/8	Supportive: Difficult population to recruit to. Use of telemedicine itself is acceptable and feasible
7	RCT (3 studies)	Gagnon, Lamothe et al. 2006 [10]	12- 30 adult palliative care patients	6/10	Partially Supportive: Proactive model can improve outcomes. Difficulties with generalising for telehome care and recruiting to an RCT in this population
8	RCT	Morgan, Craig et al. 2008 [3]	27 children with chronic heart disease	9/10	Supportive: Parents prefer to care for their child at home wherever possible. Home videoconferencing reduced anxiety scores (p =0.5)
9	Cohort	Young 2006 [11]	63 caregivers of children: 10 standard care, 16 and 34 to 2 arms of home telehealth intervention	8/11	Supportive: Home telehealth consistently reported to be an important resource that supported families. Enabled transition from hospital to home
10	Chart review	Hebert 2007 [15]	Notes from 345 adult home visits	9/11	Supportive: 43% of visits could have been done by home telehealth
11	Chart review	Doolittle 2005 [16]	Notes from 597 adult home visits	7/11	Partially Supportive: 64.5% of home visits could have been performed by home telehealth
12	Cost comparison	Doolittle 2000 [36]	2 x 3 month periods analysed (adult focus)	7/11	Partially Supportive: Home telehealth visits significantly less than in person visit ($29 vs. $129-141)
13	Quantitative	Laila et al. 2008 [13]	6 adult patients surveyed	7/11	Inconclusive: Videophones feasible and satisfactory and may have a positive effect on quality of life
14	Cohort	Demiris, Oliver et al. 2007 [28]	12 caregivers of adult palliative care patients	7/11	Partially supportive: Reported decrease in anxiety scores, but multiple confounders within study. Videophones perceived as aiding communication
15	Quantitative Survey	Washington 2008 [24]	Survey with 160 clinicians (adult focus)	11/11	Inconclusive: Moderately high acceptance, nurses and administrators more likely to use home telehealth, reluctance to use for psychosocial support
16	Qualitative	Whitten Doolittle et al. 2004 [14]	187 adult patients and caregivers	8/10	Supportive: Patients very satisfied with telehospice and wanted it used more, although some described feeling overwhelmed by technology
17	Qualitative	Demiris, Oliver et al. 2004 [25]	10 Clinicians (adult focused)	10/10	Supportive: Positive perception of telehospice, but emphasised not a replacement for actual visits
18	Qualitative / cost benefit analysis	Maudlin, Keene et al. 2006 [17]	190 adult patients	5/10	Partially Supportive: Concerns with study validity. Outcomes reported as supportive; 60% less admissions and other cost benefits with use of videophone and educational prompts
19	Qualitative	Bradford, Herbert et al. 2010 [18]	2 pediatric case studies	8/10	Supportive: Web based videoconferencing can be a simple, effective tool for supporting families at home
20	Qualitative	Doolittle, Yaezel et al. 1998 [30]	6 adult patients, 3 nurses	7/10	Supportive: Patient’s and clinicians satisfied with using videophone. Particularly helpful for rural patients
21	Qualitative	Coyle, Khojainova et al. 2002 [19]	1 adult case study	7/10	Supportive: Palliative care patients may benefit from using technology, bringing a different level of care into a patients home
22	Qualitative	Bensink, Armfield et al. 2004 [20]	1 pediatric case study	9/10	Supportive: Videophones provide a feasible method of delivering home telehealth
23	Qualitative	Olver, Brooksbank et al. 2005 [26]	7 clinicians (adult focus)	9/10	Supportive: Feasible method that provided additional support. Advantages of vision enhancing communication
24	Qualitative	Oliver, Demiris et al. 2006 [22]	2 caregivers of adult palliative care patients	7/10	Supportive: Satisfaction and technical feasibility achieved with videophones. Appears that technology was seen as a burden at the time of death
25	Qualitative	Schmidt, Gentry et al. 2011 [21]	1 adult case study	10/10	Supportive: Identified presence of non verbal communication; expression of emotion and facial expression. Videophone has potential in palliative care to provide access to non verbal communication
26	Qualitative	Cook, Doolittle et al., 2001 [27]	Interviews with 16 clinicians (adult focus)	10/10	Supportive: Barriers identified to use of telehospice program including organizational readiness and individual providers
27	Qualitative	Young 2006 [9]	Interviews with 20 caregivers of children and 2 adolescent	10/10	Supportive: Home telehealth important resource for supporting home care, provides reassurance and assists developing parental competence
28	Qualitative	Whitten 1998 [37]	Interviews with 9 clinicians (adult focused)	10/10	Supportive: Telemedicine, when used as a supplement to traditional care, may improve access issues and conceivably decrease costs
29	Mixed Methods	Oliver, Demiris et al., 2010 [23]	Interviews and questionnaires with 68 caregivers (adult focused)	10/10	Supportive: No difference seen in quality of life, but carers and staff subjectively report benefits of videophone particularly for enriching relationship and potentially to improve pain management
30	Qualitative	Johnston, Kidd et al. 2011 [31]	Focus group with 22 adult patients and 8 clinicians	10/10	Supportive: Telehealth initiatives welcomed, but should be an adjunct to clinical care rather than replacement of home visits
31	Mixed methods	Whitten, Holtz et al., 2009 [29]	25 clinicians (adult focused)	10/10	Inconclusive: Barriers not due to resources, or difficulty operating technology. Underutilization attributed to culture of organisation. Viewed as impersonal and not in alignment with goals of palliative care
32	Qualitative	Whitten, Doolittle et al., 2005 [32]	Focus groups with 61 clinicians (adult focused)	10/10	Inconclusive: Clinicians are the most important gatekeeper. Concerns regarding how telemedicine will impact on staff autonomy and financial considerations
33	Qualitative	Whitten 2005 [33]	Focus groups and interviews (adult focused)	10/10	Inconclusive: Nurses are strongest gatekeepers, other organization factors impeded use

### Evidence identified by literature and systematic reviews

Review articles were generally supportive of the use of telehealth to support palliative care patients and clinicians, but identified that more evidence was required. Bensink et al. [38] examined the use of telehealth in pediatrics and found while much had been written about its potential in the home setting, little quantitative research had been conducted. Gaikwad et al. [39] also discussed the need for more studies in this area, particularly studies that could confirm economic benefits and satisfaction with telehealth with evidence-based outcome indicators. Gaikwad reported positive outcomes attributed to telehealth, including reduction in unplanned admission rates and reduced health resource utilisation [39]. Kidd et al. [40] in their literature review of telehealth applications for palliative care in the UK found that telehealth was generally acceptable, feasible and able to increase accessibility to care. Oliver et al. [41] in a recent systematic review of the evidence for telehospice found that the evidence base was of low to medium strength in terms of quantitative studies, and that well designed Randomized controlled trials (RCT)s are required to strengthen the field. However Oliver et al’s [41] systematic review only searched for studies with the term ‘hospice’, resulting in selective reporting which missed many of the palliative care studies included in this review. Globally, the terms ‘palliative care’ and ‘hospice’ have different meanings; ‘hospice’ in some nations, Australia for example, commonly refers to a facility as opposed to the provision of care for individuals with life limiting conditions.

### Outcomes

Table 5 presents a summary of the categorized studies chronologically over the last 10 years. The studies varied widely in purpose, technology and participants. Outcomes measured included: anxiety, quality of life, costs, acceptability, satisfaction and feasibility. No two studies used the same outcome measure. The results were supportive of the use of home telehealth in palliative care situations in 67% of studies. The remaining studies were either partially supportive (6, 18%) or inconclusive (5, 15%). No studies were completely unsupportive.

**Table 5:** Level of support for home telehealth interventions for palliative care as determined by study validity and study outcomes

**Support level**	**Study year**													
	**Pre**	**00**	**01**	**02**	**03**	**04**	**05**	**06**	**07**	**08**	**09**	**10**	**11**	**Total**
Supportive	ii		i	i		iii	i	v	i	i	ii	ii	iii	22 (67%)
Partially supportive		i					i	ii	i				i	6 (18%)
Inconclusive							ii			ii	i			5 (15%)
*Total*	*2*	*1*	*1*	*1*	*0*	*3*	*4*	*7*	*2*	*3*	*3*	*2*	*4*	*33 (100%)*

### Effects on quality of life and anxiety

Quality of life is a commonly measured domain to assess the effectiveness of an intervention. Five studies, including Morgan et al. [3], Young et al. [11], Laila et al. [13], Hebert et al. [42] and Demiris et al. [28] examined the effect of home telehealth via video-consultation on quality of life and in particular, anxiety for families caring at home. Morgan and colleagues [3] focussed on children with congenital heart disease post-discharge from hospital and compared outcomes of telephone contact only with using home telehealth . They found that families who received care by telehealth had a statistically significant reduction in parental anxiety.

Young and colleagues [11] recruited families with children with complex medical needs; 44 to the intervention of ‘telehomecare’ and compared them with 10 control families. The intervention of telehomecare included remote vital sign monitoring and videoconferencing for children predominantly recovering from cardiac surgery. No statistically significant differences were found in the quality of life scores of the groups, but families subjectively reported that telehomecare provided a sense of security at an otherwise difficult time. The intervention was viewed as a successful service that enhanced the facilitation of discharge home for patients with high care needs [11]. In a qualitative report of the same study [9], the use of videoconferencing was found to consistently reduce at around three weeks after discharge. This was attributed to the increase in level of confidence families developed over time to care for their child’s needs, lessening the need to continue with videoconferencing as families ‘out grew’ the need for it. These study populations, while intrinsically different to a pediatric palliative care population, demonstrate the feasibility and acceptability of the intervention in pediatrics.

While only a small study of six subjects, Laila’s [13] study found quality of life and anxiety scores were moderately improved by the use of videoconferencing for oncology patients.

Similarly, Demiris et al. [28] conducted a small pilot study with 12 families caring for an adult patient receiving palliative care. They found that while anxiety scores did decrease, quality of life scores were not significantly changed. Any changes observed however, were likely to be a result of the intervention (the videophone) being used as a method to collect the questionnaire results. This introduces the possibility of bias into this study and therefore the results cannot be attributed to the effect of the intervention. These authors acknowledge that very few clinical calls occurred during the study period; most calls were made by the research assistant to collect quality of life and anxiety scores [28].

Hebert et al. [42] found quality of life was similar for patients randomized to receive video visits compared to usual care, concluding that care was able to be delivered by video visits.

Oliver et al. [41] examined the effect on carer quality of life of using videophones to include carers in team meetings and found no statistically significant differences between the intervention and comparator group, however subjectively caregivers and staff reported the intervention enriched their relationship.

Overall, these studies demonstrate that quality of life and anxiety may be affected positively and that no detrimental effects from the use of telehealth were observed.

### Substituting home visits with telehealth

The suitability of substituting home visits with ‘video visits’ by telehealth has been debated. Doolittle et al. [16] carried out a retrospective chart review of 597 home visits to adult palliative care patients and identified that 65 percent of these visits could have been conducted by telehealth. Hebert et al. [15] conducted a similar study, finding 43 percent of home visits could have received a ‘televisit’ instead. Demiris et al. [25,35] criticised this form of evaluating suitability for a telehealth visit as a replacement for in person home visits in two papers, arguing that telehealth is not a suitable substitute for in person visits and that there were ethical implications to consider including the medicalisation of the home environment, privacy and confidentiality, promotion of dependence and the effect of technology on the therapeutic relationships of clinicians and patients. Demiris and colleagues went on to acknowledge that while telehealth has been integrated with palliative care successfully in some institutions, more evidence is required to evaluate its effectiveness [25]. Johnston et al. [31] likewise found that telehealth initiatives have been welcomed by patients and carers but the caveat was that telehealth should be used as an additional/complementary tool, not as a substitute or replacement to usual care.

### Economic considerations

From the economic perspective, Doolittle et al. [36] found there were significant savings to be made if ‘video visits’ were used instead of home visiting. Maudlin et al. [17] also reported cost benefits associated with videoconferencing and text messaging to prompt and educate adult patients regarding self care strategies. Additionally Maudlin and colleagues reported a reduction in admissions to hospitals which was attributed to the use of these telehealth initiatives [17].

Gagnon [10] however argued that it was necessary to acknowledge the economic limits of telehealth in cases where services supplement, rather than replace visits. If video-conferencing is an adjunct to usual care, supplementing or improving care when home visiting is not possible, then any cost comparison of video conferencing compared to in person visits are irrelevant as home visiting is not intended to be replaced by video-conferencing. This makes it difficult to quantify economic benefits and perform accurate cost analysis as the videoconferences are an adjunct rather than a replacement to usual services. Indeed, it may be found that providing telehealth services actually increase rather than reduce costs for health care providers.

For families, telehealth services have been reported to reduce the cost associated with travel and time attending appointments, and reduce anxiety for caregivers which has a potential although unquantified economic value [39]. However, while these savings are beneficial to individuals, they are difficult to use as a justification for services by providers of health care.

### Barriers to use

Understanding the barriers to telehealth is an important consideration for any providers of a service. In a series of studies, Whitten et al. [29,32,33,37]and Demiris [28], identified that clinicians act as ‘gatekeepers’ to using telehealth services and that barriers to using technology were related to the culture within health care settings. Whitten discussed the notion that telehealth was viewed as impersonal, lacking in human touch, and that in a palliative care setting where the goal is to comfort patients and families, some clinicians viewed telehealth negatively [29,32]. Additionally, Whitten found that there were issues relating to financing and re-imbursement for travel; nurses may prefer the financial rewards associated with home visits and resent an initiative that will reduce their potential income [33,37]. Other barriers postulated by Hebert [44] and Oliver et al. [41] were that the slow uptake of telehealth in the home care setting for palliative care is due to the lack of evidence and difficulties quantifying economic benefits.

### Feasibility, acceptability and satisfaction with telehealth

There were a large number of qualitative papers (18 in total) that described the use of telehealth in home care situations. Six papers [18-20,22,26,30] presented case study examples that found that telehealth applications were well received by patients and clinicians. These applications were perceived as being a helpful and feasible method of delivering care, particularly for increasing access to care for families who were otherwise isolated by geography or because they were house bound. Oliver et al. [22] presented two case studies in which both ceased using telehealth as the patient’s condition deteriorated. Oliver concluded that it appeared that the technology was seen as a burden at this time by family members. This adds weight to the concept discussed by Young [11] that video consultations may reach a threshold where their usefulness ceases.

Washington et al. [24] in their survey of 160 health care professionals found that nurses and administrators were more likely to accept telehealth compared to social workers and chaplains. This would indicate that for psychosocial interventions, telehealth is perhaps not as readily accepted. Whitten [29] and Demiris [25] found that some clinicians didn’t like telehealth as it was perceived to limit the ability to communicate on a personal level. Schmidt et al. [21] however challenged the perception that telehealth lacks the ability to communicate effectively and empathetically, demonstrating nonverbal communication was conveyed during videophone interactions and that emotional communication was present. Other qualitative papers presented small observational studies with generally positive results that are difficult to generalise to wider populations. In terms of systemic change, these studies have been too small to have influenced uptake of home telehealth.

### Organisational readiness

Cook et al. [27] interviewed stakeholders who were involved in the multiple studies undertaken by Doolittle et al. [16,30,36] and provided strategies to ensure the success for a telehealth application in the palliative care population. Four key elements were proposed: the project coordinator must be fully engaged with participants (clinicians and patients); a seamless delivery process should be defined by the co-ordinator; a patient centred approach is required, and champion clinicians who support the project must be identified. Cook [27] explained that it is ultimately clinicians who will drive the use of a telehealth system and without their support and motivation, telehealth in this population is not likely to succeed. Similarly Gagnon [10] identified that it is organisational readiness not scientific evidence acquired from research studies that are needed in order for ‘home telecare’ to be widely adopted.

### Study design considerations

Most studies were descriptive and those that did involve an RCT had small sample sizes or otherwise lacked scientific rigour. Bensink et al. [44] identified inherent difficulties with running an RCT in the pediatric palliative population. Bensink’s study, which aimed to evaluate home telehealth by measuring changes in caregiver quality of life, was abandoned twice due to poor recruitment. It was assumed that the failures were due to the perceived burden and intrusiveness of the study design at a difficult time [44]. In 2004, Hebert et al. [43] reported planning a similar study, albeit in the adult palliative care population and aimed to recruit 320 participants. Results were reported in 2006 [42]; due to changes in referral patterns to home care services, recruitment was difficult and only 44 participants were randomized. Hebert et al. reported that similar quality of care was achievable by video visits, but that due external factors such readiness to use telehealth, video visits were unlikely to be incorporated into routine practice with this population. Gagnon et al. [10] evaluated three telehealth studies which focused on vulnerable populations and acknowledged that success of telehealth projects in this population are hindered by the need to recruit a ‘critical mass’ large enough to prove effectiveness. Another factor Gagnon identified as influencing the success of a study in telehealth in palliative care included having a clinician involved in the study design.

## Discussion

This review identified 33 studies which were relevant to the application of home based telehealth to support palliative care families, only six of which were specific to pediatrics. The results from these studies were generally supportive of this application; however to successfully utilize this form of communication, several areas were identified which require careful consideration.

Historically, studies have proved difficult in this area due to low recruitment and a subsequent inability to show effectiveness. Measuring the effect of telehealth in palliative care is challenging as outcome measures such as quality of life are not easily attributable to the telehealth intervention. Additionally there is debate within the literature regarding the role of home telehealth applications [10,29,31]. There are however, a number of small but successful studies that demonstrate home telehealth to be a useful and feasible method of providing support to families. These studies have found a reduction in anxiety scores [3,28], enhanced communication between clinicians and families [9,20,31], and a decrease in unplanned admission rates to hospitals and health care utilisation [39]. Telehealth was also seen to be a cost and time effective method of delivering care [17,36]. Despite these positive findings, telehealth is not widely used in palliative care home settings. Reasons given include clinician preference [29] and difficulties establishing the effectiveness of services [10,39]. There also remains the possibility of other, yet undefined barriers.

There are inherent challenges of conducting research in palliative care and the importance of careful consideration to methodology and study design cannot be overstated. In the pediatric palliative care population, challenges are even greater: ethical issues around consent and assent of the child need to be considered; the child may not be aware that they are dying; and caregivers may be struggling to come to terms with their child’s inevitable death and may not wish to participate in research at this time. Additionally the focus of care for many children remains treatment orientated as opposed to palliation and there may be only a small window available for potential recruitment to a research study [45]. A randomized controlled trial therefore, is not the most feasible or ethical design in many cases. Observational studies are useful in providing evidence and collaborative efforts may improve the ability to recruit the numbers required for scientific rigour. A practical framework for understanding studies in this area may be useful, integrating the findings from this review with appropriate evaluation of home telehealth services.

### Framework for home telehealth in palliative care

**Figure 2:**
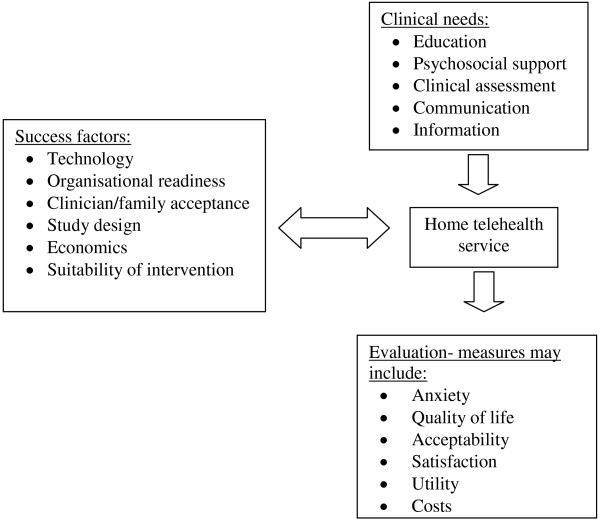
Practical framework for home telehealth in palliative care.

Using the areas identified in this review, a framework [46] was developed to explain the relationships between: clinical needs, factors which enable or hinder home telehealth, and evaluation measures (Figure 2). Fulfilling patient and family needs could be used to evaluate the effectiveness of a telehealth intervention, assessed either quantitatively or qualitatively, depending on the individual study design or evaluation. Thus a focus on evaluating specific criteria identified as a need by the family or clinician, such as effective symptom management, or met educational needs may provide evidence of effectiveness more easily attributable to a telehealth intervention than measures of quality of life or anxiety.

### Gaps in the literature

This review identified examples of home telehealth applications for various populations requiring complex care at home: pediatric oncology [18,20,44], pediatric cardiac [3,9,11], adult oncology [13,28] and adult palliative care [10,26,40,42]. What remains unknown is whether within these populations, there are intrinsic differences that affect the acceptance or use of telehealth applications. Variables such as goals of care, access to alternative modes of care, perceived need for care, comfort with using technology and even the physical location of the technology within either the home or health care facility may also influence use. For home telehealth in palliative care to be established as a viable method of facilitating care, further studies are required to build on the evidence base. In pediatric palliative care particularly, there is little known about barriers, benefits and limitations, factors influencing use and the economic implications of telehealth applications to support home care. Further studies in these areas, with careful attention to the logistical and ethical issues of conducting research with this vulnerable population are needed. However any research conducted needs to be carefully planned, with attention to partnership with pediatric palliative care clinicians, minimisation of burden and unnecessary procedures, easily definable inclusion criteria; and flexible data collection methods [45].

### Limitations

Some articles may have been missed when undertaking the search. Due to time constraints the grey literature was not searched. Given the high number of papers that report home telehealth as an effective means to provide support, publication bias may be present in the articles included in this review. Additionally there was a high degree of heterogeneity in the included studies and because of the small numbers of articles, it was necessary to combine analysis of studies with different study designs.

## Conclusion

Telehealth has been demonstrated to be a feasible and effective method of delivering information, education and support. The full potential of telehealth applications has not been realised and the use of telehealth to support palliative care patients being cared for at home requires further investigation. Over the last decade a number of studies have attempted to measure the outcomes of telehealth applications in the home setting for this population. The inability of these studies to establish effectiveness demonstrates the difficulty of measuring an effect of an intervention such as telehealth in palliative care. Despite these limitations, there are numerous examples of individual case studies where telehealth has successfully been used for its intended purpose to support families in their homes, and also some suggestions of the limits of this form of technology. Home-based telehealth has the potential to improve services and outcomes for families. Further research is therefore warranted to establish the role of home telehealth in the pediatric palliative care setting.

## Competing interests

The authors declare that they have no competing interests.

## Authors’ contributions

AS, NR, JY and NB conceived and participated in the design of the study. NB conducted the search of the literature, preliminary analysis of data and initial draft. NR reviewed the methodology and analysis. All authors contributed to, and read the final manuscript.

## Pre-publication history

The pre-publication history for this paper can be accessed here:

http://www.biomedcentral.com/1472-684X/12/4/prepub
